# Evaluation of Periodontal Tissues in Growing Patients with Bilateral Cleft Lip and Palate. a Pilot Study

**DOI:** 10.34763/devperiodmed.20172102.154161

**Published:** 2017-08-11

**Authors:** Beata Wyrębek, Dorota Cudziło, Paweł Plakwicz

**Affiliations:** 1 Department of Periodontology, Medical University of Warsaw, Warsaw Poland; 2 Head of Department of Maxillofacial Orthopaedics and Orthodontics, Institute of Mother and Child in Warsaw, Warsaw Poland

**Keywords:** bilateral cleft, mucogingival conditions, oral hygiene, periodontal status, higiena jamy ustnej, obustronny rozszczep, status periodontologiczny, warunki śluzówkowo-dziąsłowe

## Abstract

**Aim:**

To evaluate the periodontal status, mucogingival parameters and oral hygiene in growing patients with bilateral cleft lip and palate.

**Material and methods:**

Assessment was performed in 15 patients aged 6 to 18 years with a bilateral cleft. Records included probing pocket depth, clinical attachment level, keratinized gingiva, recession, vestibule depth, biotype, type of fraena, dental plaque and bleeding.

**Results:**

The mean scores of pocket depth were: 1.9 mm for central incisors, 1.6 mm for lateral incisors, 1.7 mm for canines, 2.0 mm for first premolars. There were only a few teeth with minimal attachment loss (1 mm). Gingival recessions were not recorded. High scores were recorded for the hygiene indicator, especially on the buccal, mesial and distal surfaces. Due to soft and hard tissue malformations, it was difficult to precisely assess the biotype and keratinized gingiva. However, keratinized gingiva was narrower near the teeth adjacent to the cleft. Similarly, the vestibule was shallower in this area. In 12 out of 15 children it was impossible to define the type of labial fraenum.

**Conclusions:**

Evaluation of the periodontal status is important for successful comprehensive rehabilitation in cleft patients. Specific features of hard (alveolar process) and soft tissue (scars, unusual fraena) malformations caused by the cleft and previous surgical procedures have functional and morphological implications. Narrower gingiva and a shallower vestibule in the presence of dental plaque and bleeding are unfavourable conditions to maintain a healthy periodontium. It is essential to include periodontal assessment and preventive treatment to a comprehensive approach as early as possible.

## Streszczenie

**Cel:**

Celem pracy była ocena stanu przyzębia, parametrów śluzówkowo-dziąsłowych oraz higieny jamy ustnej u pacjentów z obustronnym rozszczepem wargi i podniebienia w okresie wzrastania.

**Materiał i metody:**

Badaniu poddano 15 pacjentów wieku 6-18 lat z obustronnym rozszczepem wargi i podniebienia. Badanie periodontologiczne obejmowało pomiar głębokości szczelin dziąsłowych, poziomu przyczepu łącznotkankowego, szerokości dziąsła skeratynizowanego, recesji, głębokości przedsionka, ocenę biotypu i typ przyczepu wędzidełek. Obliczono również wskaźnik płytki nazębnej oraz krwawienia.

**Wyniki:**

Średnie wartości szczelin dziąsłowych wynosiły: 1,9 mm dla zębów siecznych przyśrodkowych, 1,6 mm dla zębów siecznych bocznych, 1,7 mm dla kłów, 1,8 mm dla pierwszych zębów przedtrzonowych. Zanotowano tylko nieznaczną utratę przyczepu łącznotkankowego przy nielicznych zębach (≤1 mm). Nie odnotowano obecności recesji dziąsłowych. Stwierdzono wysoką wartość wskaźnika płytki nazębnej, szczególnie na mezjalnych, dystalnych i wargowych powierzchniach badanych zębów. Z powodu deformacji tkanek miękkich i twardych trudno było precyzyjnie ocenić biotyp i szerokość dziąsła. Odnotowano jednak mniejszą szerokość dziąsła przy zębach graniczących z rozszczepem. Również przedsionek jamy ustnej był w spłycony w tym obszarze. U 12 z 15 dzieci niemożliwe było ustalenie typu wędzidełka wargi górnej.

**Wnioski:**

Badanie periodontologiczne jest istotne dla pełnej rehabilitacji pacjentów z rozszczepem. Deformacje tkanek twardych (ubytki kości) i miękkich (blizny, fałdy błony śluzowej, zniekształcone wędzidełka, dodatkowe więzadła) spowodowane przez rozszczep, jak również przez wcześniejsze zabiegi chirurgiczne, wpływają na funkcję i estetykę tego obszaru. Mniejsza szerokość dziąsła i płytszy przedsionek przy zębach graniczących z rozszczepem, szczególnie w obecności płytki bakteryjnej i krwawienia, są niekorzystnymi warunkami do utrzymania zdrowych tkanek przyzębia. Stąd należy włączyć okresowe badanie periodontologiczne w wielospecjalistyczne leczenie pacjentów z rozszczepem.

## Introduction

Cleft lip, alveolus, and palate are congenital malformations created in the early phase of embryogenesis [1]. The aetiology is unknown, but it is considered to be complex. Some cleft lips and palates have a genetic origin, others may be caused by environmental factors [2]. Children with a cleft require multidisciplinary treatment due to problems during feeding, speaking, listening, as well as frequent ear infections and psychosocial issues [2,3].

Although surgery is performed in the first few months of life, most children have a deficiency of soft tissues, bone volume, malformation and/ or lack of teeth at the cleft site [4]. These features, as well as side effects of surgeries itself (scars, unusual fraena attachments), cause obstacles during orthodontic and restorative treatment and negatively influence aesthetics. Children with clefts need orthodontic treatment, which provides alignment and stabilization of the teeth after reconstructive procedures [5]. The timing of bone graft placement is an important issue. The most commonly accepted procedure is secondary bone grafting (during the mixed dentition period) to provide alveolar bone support for the erupting teeth. Bone grafting in adults is associated with a higher risk of graft failure due to changes in the healing potential caused by the age of the patient [5, 6]. According to some studies, early performance of secondary gingivoalveoloplasty combined with hard palate closure at the age of 18-36 months makes it possible to avoid bone grafting in the future. Permanent tooth eruption occurs at a normal rate, without the need for secondary alveolar bone grafting [7]. The one-stage surgery method seems to improve anatomical conditions in the craniofacial area and enables better further development [8]. Bone grafting performed after eruption of the canine may lead to insufficient marginal bone height and gingival recession at the teeth adjacent to the cleft . In addition, the vestibular flap technique used to cover the bone grafts, may result in soft tissue scars and shallow vestibule, which demand additional surgical procedures [5, 6, 9].

Moreover, it is important to emphasize that the boundary structures of the oral vestibule have not been formed properly and should be reconstructed as well. On the other hand the vestibular flap technique performed to cover bone grafts in early childhood may result in a scarred and shallower vestibule with loose folds of mucosa [9]. This problem may have functional, hygienic and aesthetic implications. The normal function and appearance of the lip is only possible when the lip can freely move over the teeth during speaking and smiling. The depth of the vestibule is important to protect against infection and functional muscle forces created during eating. In case of an inadequately formed vestibule, additional stress is placed on the attached gingiva in patients with bilateral cleft . This could potentially cause mucogingival (gingival recessions) and periodontal problems (periodontal disease). Moreover, a malformed vestibule may have a negative influence on dentition status and on the maintenance of oral hygiene. The normal depth of the oral vestibule enables prosthodontic and orthodontic treatment [7, 9, 10].

Furthermore, it is a well-known fact that the smile is characterized by the relationship between the teeth, lips, and gingival tissue. Dental evaluation includes external features, such as: face profile, smile and lip line, teeth and gingiva exposure during smiling and intra-oral conditions, such as the number of teeth, gingival architecture, biotype, tooth status [11, 12]. Planning oral rehabilitation with dentures or implants (usually including orthodontic treatment) depends on the extent of exposure of the gingiva, especially during speaking and smiling. That is why the healthy appearance of the gingiva is important in smile aesthetics [12].

The above reasons justify why periodontal evaluation is directly related to the aesthetic and functional rehabilitation of patients with clefts. Evaluation of periodontal conditions is often neglected or insufficiently documented, since the patients’ major problems are related to reconstructive treatment and quality of life [11, 12]. As a result, there are very few studies regarding the periodontal status in cleft patients.

The aim of the study was to evaluate the periodontal status, mucogingival parameters and hygiene indices in growing patients with bilateral cleft lip and palate and to compare the results with other studies.

## Material

The sample consisted of consecutively selected patients with complete bilateral cleft lip and palate, who were treated in the Orthodontic Department of the Institute of Mother and Child in Warsaw, Poland. 15 Caucasian individuals, aged from 6 to 18 years (nine females and six males) with mixed or permanent dentition were examined. The measurements were performed on eight anterior teeth in the maxilla (102 teeth in total). In seven patients both maxillary lateral incisors were missing, in other four patients one lateral incisor was missing. All the patients underwent reconstructive surgery of the cleft lip, alveolar process, hard palate and soft palate. Due to maxillary hypoplasia, all the patients were treated with removable or permanent orthodontic appliances. The aim of orthodontic treatment was to achieve a favourable condition for three-dimensional development of the maxilla and to create space for the eruption of permanent teeth. Records were taken between July and November 2015. Exclusion criteria were as follows: the presence of systemic syndromes, hemi- or paraplegia of the face, conditions or/ and medication that could influence bone or soft tissue metabolism. after the examination of the patients, instructions concerning oral hygiene were given depending on individual needs.

## Methods

Clinical examination of the patients was performed using a periodontal probe (Hu-Friedy, PCP UNC 15, calibrated to 1 millimetre). Periodontal examination included records for:

Probing pocket depth (PD) measured as the distance from the gingival margin to the bottom of the gingival sulcus. Assessment was carried out at six sites: distolabial, labial, mesiolabial, mesipalatal, palatal and distopalatal.

Clinical attachment level (CAL) measured as the distance from the cementoenamel junction (CEJ) to the bottom of the sulcus. Assessment was carried out at the same six sites as PD.

Gingival Recession (GR) calculated as a distance from CEJ to the gingival margin in case of root exposure. If CEJ was covered by gingiva it meant that there was no recession.

Keratinized gingiva (KG) measured at the labial (midfacial) surface of the tooth as the distance from the gingival margin to the mucogingival junction.

Vestibulum oris depth (VOD) measured as the distance from marginal gingiva to the highest point of the vestibule in relaxed muscle position. The measurements were taken at labial midfacial surfaces of each tooth.

The presence of dental plaque was assessed by Plaque Control Record (PCR) according to the O’Leary Plaque Index [13] at four aspects of the tooth: labial, palatal, mesial and distal. A record was considered positive if there was dental plaque on the probing surface. The index was calculated as a percentage, by dividing the number of surfaces containing plaque by the total number of available surfaces and multiplied by 100. The mean PCR scores for every tooth surface and group of teeth were calculated.

Bleeding on probing (BoP) was evaluated according to Ainomo and Bay [14] at six aspects of the tooth: centrolabial, centropalatal, mesiopalatal, distopalatal, mesiolabial, distolabial. A periodontal probe was inserted at the bottom of the sulcus and was moved along the tooth surfaces. If bleeding was noticed, then the examined site was considered positive. The BoP index was calculated in the same way as PCR and mean scores were given as a percentage.

The gingival biotype was assessed separately for the maxilla and mandible. The soft tissue biotype was classified as thin or thick. Thin biotype was recorded when thin and fragile gingival tissue and a narrow band (width) of keratinized gingiva were detected. Thick biotype was recorded when thick, dense and fibrotic soft tissue and a wide band of keratinized gingiva were detected.

The type of fraena of the upper and lower lips according to the Placek classification [15] was recorded (mucosal, gingival, papillary and penetrating papilla). The presence of additional ligaments and folds, which were results of clefts and reconstructive surgeries, were also recorded.

## Results

102 teeth in ten patients were assessed. The data regarding PD, CAL, GR, KG, VOD, PCR, BoP, biotype and labial fraena are summarized in tables. Mean scores for PD for a particular group of teeth were: 1.9 mm for central incisors, 1.6 mm for lateral incisors, 1.7 mm for canines, 2.0 mm for first premolars (tab. I). There was only minimal CAL loss, which did not exceed 1 mm (tab. II). Gingival recessions were not observed. High scores for dental plaque (PCR) were recorded on the labial and interproximal (mesial and distal) surfaces contrary to palatal tooth surfaces (fig 2). The mean scores for particular groups of teeth were: 49.5% for central incisors, 38.5% for lateral incisors, 43.5% for canines and 37% for first premolars (tab. III). The scores for bleeding (BoP) were as follows: 32% for central incisors, 24% for lateral incisors, 27% for canines and 19% for first premolars (tab. II).

**Table I:** Mean scores for pocket depth PD (in millimetres) for each measurement site (distal, central and mesial) on the labial and palatal side of teeth and mean PD results for each group of teeth (n=number of teeth, N-number of tooth surfaces). Tabela I. Średnie wyniki głębokości szczelin dziąsłowych SD (w milimetrach) dla każdego miejsca pomiaru (dystalnie, centralnie i mezjalnie) na wargowej i podniebiennej stronie zębów oraz średnie wyniki SzD dla każdej grupy zębów (n = liczba zębów, liczba N powierzchni zębów).

Tooth group: *Zęby*	Cl (central Incisor), *Siekacze przyśrodkowe* n=30, N=180			LI (lateral incisor), *Siekacze boczne* n=12, N=72			C (canine), *Kły* n=30, N=180			FP (first premolar), *Pierwsze przedtrzonowce* n=30, N=180		
Surface: *powierzchnia*	Distal *środkowa*	Central *dystalna*	Mesial *mezjalna*	Distal *dystalna*	Central *środkowa*	Mesial *mezjalna*	Distal *dystalna*	Central *środkowa*	Mesial *mezjalna*	Distal *dystalna*	Central *środkowa*	Mesial *mezjalna*
Labially *wargowa*	1.9	1.7	2.1	1.7	1.1	1.7	1.9	1.2	2.0	2.3	1.5	2.1
Palatally *podniebienna*	2.1	1.9	1.9	1.9	1.6	1.8	1.8	1.5	2.0	2.2	1.5	2.2
Mean PD *średnia* (range); Odch. SD stand. SD	1.9 (1-3); 0.7			1.6 (1-3); 0.6			1.7 (1-3); 0.6			2.0 (1-4); 0.7		

**Table II:** Mean scores for clinical attachment level CAL (in millimetres) and mean scores for BoP (in %) for each tooth group (n=number of teeth, N-number of tooth surfaces). Tabela II. Średnie wyniki poziomu przyczepu łącznotkankowego − PŁ (w milimetrach) i średnie wyniki dla wskaźnika krwawienia − WK (w%) dla każdej grupy zębów (n = liczba zębów, liczba N powierzchni zębów).

Tooth group: *Zęby*	CI (central Incisor) *Siekacze przyśrodkow*e n=30, N=180	LI (lateral incisor) *Siekacze boczne* n=20, N=72	C (canine) *Kły* n=30, N=180	FP (first premolar) *Pierwsze przedtrzonowce* n=30, N=180
Mean CAL in mm *Średni poziom P£* (range); SD	0.03 (0-1), 0,2	0 0.0; 0.0	0.025 (0-1); 0.2	0.03 (0-1); .,2
Mean *Średni* BoP *WK* in %	32%	24%	27%	19%

**Table III:** Mean scores for plaque control record - PCR (in %) for each tooth group (n=number of teeth, N-number of tooth surfaces). Tabela III. Średnie wyniki dla wskaźnika płytki bakteryjnej − PB (w%) zębów dla każdej grupy (N = ilość zębów, N − liczbę powierzchni zębów).

Tooth group: *Zęby*	CI (central Incisor) *Siekacze przyśrodkowe* n=30, N=120	LI (lateral incisor *Siekacze boczne* n=12, N=48	C (canine) *Kły* n=30, N=120	FP (first premolar) *Pierwsze przedtrzonowce* n=30, N=120
Mean *Średnia* PCR *PB* in %	49.5	38.5	43.5	37.0

KG was narrower near the teeth adjacent to the cleft sites. Mean KG at the lateral incisors was 1.3 mm, at the canines 1.9 mm, while at the central incisors KG it was 5.3 mm and 2.7 mm at the first premolars (tab. IV). The mean VOD was also lower in the cleft area. For lateral incisors it was 3.4 mm and 3.8 mm for canines. VODs for premolars and central incisors were higher (5.4 mm and 7.9 mm respectively) (tab. IV).

**Table IV:** Mean scores for keratinized gingiva - KG and for vestibulum oris depth − VOD (in millimetres) on the labial surfaces of tooth groups (n=number of teeth, N-number of tooth surfaces). Tabela IV. Średnie wyniki w szerokości dziąsła skeratynizowanego DzS a oraz dla głębokości przedsionka GP (w mm) po wargowych powierzchni zębów grupy (n = ilość zębów, N − liczbę powierzchni zębów).

Tooth group: *Zęby*	CI (central Incisor) *Siekacze przyśrodkowe* n=30, N=30	LI (lateral incisor *Siekacze boczne* n=12, N=12	C (canine) *Kły* n=30, N=30	FP (first premolar) *Pierwsze przedtrzonowce* n=30, N=30
Mean KG in mm *Średnia DzS*	5.3	1.3	1.9	2.7
Mean *Średnia* VOD in *GP* mm	7.9	3.4	3.8	5.4

**Fig. 1:**
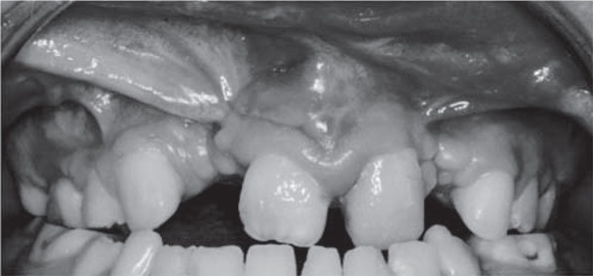
Intra-oral photograph of 13-year-old patient with bilateral cleft and accompanying agenesis of teeth 15, 12, 22. Figure presents unusual mucosal folds and fraena in the upper part of the oral vestibule. Scars within soft tissue are side effects after previous surgeries that aimed to reconstruct tissues of the palate and alveolus as well as the soft tissues of the upper lip. Ryc. 1. Zdjęcie wewnątrzustne 13-letniego pacjenta z obustronnym rozszczepem i towarzyszącą agenezą zębów 15, 12, 22. Widoczne nietypowe fałdy błony śluzowej oraz wędzidełka wargi górnej. Blizny w tkance miękkiej są skutkami ubocznymi po poprzednich zabiegach, które miały na celu odtworzenie ciągłości tkanek podniebienia, wyrostka zębodołowego oraz oraz wargi górnej.

**Fig. 2:**
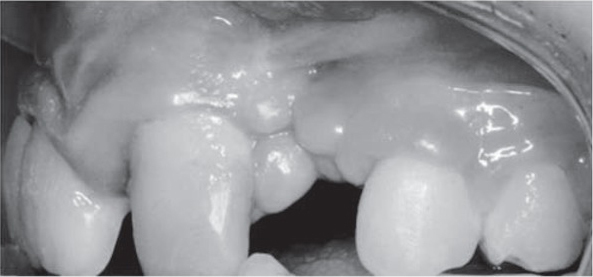
Lateral intra-oral photograph of the patient shows the accumulation of dental plaque on tooth surfaces adjacent to the cleft site and inflammation of marginal gingiva. Additionally the complexity of soft tissue morphology can be seen at the site of lateral incisor agenesis that corresponds to the cleft area. Ryc. 2. Boczne, wewnątrzustne zdjęcie pacjenta pokazuje gromadzenie się płytki nazębnej na powierzchniach zębów w sąsiedztwie szczeliny rozszczepu oraz zapalenie dziąsła brzeżnego. Dodatkowo złożoność morfologii tkanek miękkich można zaobserwować w miejscu agenezy siekacza bocznego, która odpowiada obszarowi rozszczepu.

In 12 out of 15 patients it was impossible to define the type of labial fraenum in the maxilla. Two patients had a mucosal type of fraenum attachment and two patients had mucosal types. Due to soft tissue malformations (scars, unusual fraena attachments, mucosa folds, additional ligaments), it was difficult to assess the type of gingival biotype in the maxilla (fig. 1, 2). In the mandible nine patients presented thin biotype and six presented a thin biotype.

## Discussion

There are only a few studies on periodontal conditions in growing patients with cleft s, In fact the periodontal status may have important implications in the comprehensive treatment of these individuals [16, 17, 18, 19]. Alveolar process deficiency has a negative impact on soft tissue appearance, causing functional and aesthetic problems in this area. Additionally, combined deficiencies of hard and soft tissues are particularly difficult to treat [4, 6, 7, 8]. Most authors are consistent that patients with clefts are at an increased risk for the development of periodontal disease and carious lesions. However, the data are still limited. Other authors claim that there are no major differences between the teeth in the cleft and the non-cleft sites regarding periodontal status [20]. Patients with clefts are at high risk of progress of periodontal disease if no supportive periodontal therapy is provided in their early childhood. It has already been presented that adult patients with cleft s, high plaque score and gingival inflammation had more periodontal tissue destruction. Moreover, the risk of periodontal disease and the level of tissue disorders increased with age [21]. The aim of this study was to assess the periodontal status in the area of bilateral cleft in growing patients.

According to Perdikogianni and co-workers [22], teeth in the cleft area had higher pocket probing depths compared with the corresponding teeth in the control group, although pocket depths were up to 3mm, which according to the authors was considered within normal limits. Quirynen and co-workers [20] found only an insignificant increase in the probing depth of the teeth in the cleft site, when compared to the non-cleft site. In the study presented there were no gingival pockets deeper than 4 mm. Pockets deeper than normal (>2 mm) but without CAL loss may indicate gingival inflammation or hyperplasia, which results in coronal displacement of the gingival margin. It may be due to the presence of an orthodontic appliance or inadequate plaque control. Established poor oral hygiene may lead to periodontal inflammation with bone loss, because of more pathogenic bacteria subgingivally [23]. The presence of deeper pockets without gingiva inflammation could lead to incomplete tooth eruption, which was also found in some of the patients evaluated.

The few studies that analysed the periodontal status of cleft patients showed a high incidence of plaque and bleeding on probing and a high level of periodontal attachment loss [21, 24]. In our study there was only minimal CAL loss, which did not exceed 1 mm. Since periodontal parameters worsen with age, this difference could be due to the young age of the patients in the group evaluated. Furthermore, Bragger and co-workers reported that alveolar bone loss was more advanced at a cleft site compared with control, although the clinical attachment level was similar on both sites [25]. These findings suggested the presence of a longer connective tissue attachment in the cleft regions. Reduced bone support might, however, cause several problems in the future in case of inadequate plaque control. Thus, professional evaluation and treatment, if required, is essential for maintaining the periodontal health of these patients.

According to Almeida and co-workers, the prevalence and severity of gingival recessions increased with age [26]. According to this study, the cleft area did not present a higher prevalence and severity of gingival recession when compared with non-cleft patients. In our results gingival recessions were absent. The main reason could be the thick structure of keratinized gingiva caused by scars after surgery that protected gingival margins against root exposure. Areas with a narrow zone of keratinized gingiva may have a similar level of resistance to potential root exposure as gingiva with a wide zone in the case of the presence of the adequate thickness of the gingiva [27]. The study presented showed that even though the keratinized gingiva near the cleft was narrow, there were no gingival recessions at adjacent teeth. However, one must remember that only in patients maintaining proper plaque control, the lack of a wide zone of gingiva would not result in clinical attachment loss and recessions [25, 26, 27]. Almeida and co-workers claimed that factors such as tooth position in the dental arch, the presence of fraena or scars, the absence of keratinized mucosa and traumatic tooth brushing might increase the prevalence of recession, however in their study the most affected teeth were not adjacent to the clefts [28]. In another study the authors concluded that the prevalence of recession in teeth close to the cleft was higher than the same teeth in patients without clefts, although the recessions were not severe [29]. Teeth at cleft sites may present higher occurrence of gingival recession in the future, due to reduced bone support, as well as to the low quantity of mucosa [26, 28, 29].

Moreover, the reason for the lack of root exposure in our study could be related to the young age of the patients. The prevalence of gingival recession depends on the level of oral hygiene, with a frequency between 12% and 19% in children, 15.5% to 54.5% in young adults, and 57.7% to 100% in older individuals. The high prevalence in older groups was related to the longer exposure of their teeth to etiologic factors of recessions [21]. There is a potential risk that due to the presence of aetiological factors of recessions, children with clefts may develop gingival recession in the future [28].

In patients with a bilateral cleft , the boundary structures of the oral vestibule are malformed [9, 10]. Moreover, as a result of previous surgeries, such as cheiloplasty and bone grafting, there might be even more limited space for a toothbrush, which combined with gingival folds favour food debris accumulation. An inadequately formed vestibule in cleft patients causes additional stress from the malformed lips on the marginal gingiva on the labial teeth surfaces. This could lead to recession, periodontal disease and compromise the dentition status. A normal vestibule facilitates prosthodontic and orthodontic treatment [9]. In the study presented the vestibule was shallower near the teeth adjacent to the cleft (second incisors and canines) in comparison to other teeth that were evaluated. It was difficult to assess the vestibular depth, because of mucosa folds, fraena, as well as teeth malposition. For that reason it could not be determined whether the presence of the cleft alone decreased the vestibular depth, or whether it resulted from the presence of different factors.

The presence of an orthodontic appliance, the sti$ ness of the upper lip due to scar formation, crowding and malformation of the teeth may hamper optimal oral hygiene [16, 18]. soft tissue folds and deformities of mucosa make tooth brushing difficult. These areas constitute a habitat for food debris and bacteria accumulation and consequently increase the risk of periodontal infection and caries [20, 22, 23]. The results of the study presented indicated that oral hygiene was not optimal in patients (fig. 2). These data were consistent with other studies that also recorded high scores for dental plaque in cleft patients [20, 22, 23]. However, the differences between cleft and non-cleft sites were small in the study assessing oral hygiene in unilateral cleft patients [20]. It suggests that not only mucosa malformation but also young age and orthodontic appliances are the reasons for inadequate plaque control. Furthermore, according to Bragger and co-workers, due to inadequate oral hygiene, adult patients with clefts demonstrated early signs of periodontitis with furcation involvement in most of cases [30]. This is consistent with another study that showed that over a 14-year period cleft sites in subjects with high plaque and gingival inflammation underwent more periodontal tissue destruction than control sites [21].

The study presented showed irregularities of the gingiva margin and soft tissue at the teeth adjacent to the cleft . There were scars and mucosa folds in the vestibule, however pull-syndrome was not present et the marginal gingiva (fig. 1, 2). There were also atypical upper labial fraena and the biotype was difficult to assess. These characteristics, which had been observed also by other authors, were present due to the cleft itself, but also as a result of surgical treatment [9, 11, 18].

## Conclusions

The results of the present study indicate that:

1. Malformations of hard and soft tissue caused by the cleft itself and previous surgical procedures have a negative influence on periodontal parameters in the cleft area.
2. Narrow gingiva and shallow vestibule at the cleft site in the presence of dental plaque do not favour maintenance of a healthy periodontium. This corresponded with increased bleeding at some teeth adjacent to clefts.
3. It seems essential to include regular periodontal examination and prophylaxis into comprehensive treatment in cleft patients. Further assessment of a large-sized group is necessary to establish the preventive protocol in multidisciplinary treatment.

## Instructions for Authors in *Developmental Period Medicine*

1. ***Developmental Period Medicine*** is a journal published by the Institute of Mother and Child in Warsaw. It is indexed in MEDLINE/PUBMED. The journal publishes original research studies, reviews, clinical case studies, public health studies and communications, both in Polish and in English. It is preferable for the texts to be submitted in English.
2. Manuscripts should be sent to the editors’ address in two copies of single-sided computer printouts, on A4 format paper with a margin of 4 cm on the left side and double space between the lines. Authors are asked to make the text available on electronic media and specify the word processor used, preferably be Word for Windows. Texts can be sent by e-mail.
3. Original articles, together with tables, figures and references should not exceed 21 600 characters (12 pages), reviews 36 000 characters (20 pages).
4. The front page of the manuscript should contain:
  – the title of the article in English and Polish
  – the author(s)’ forename(s) and surname(s)
  – the authors’ affiliation
  – their scientific titles
  – the first letter of the first name and the surname of the head of the institution.
5. The text of original research studies must have the following structure: introduction, aim, materials and methods, results, discussion, conclusions (which cannot be a summary of the paper). Abbreviations must be preceded by the full text when first used. In research studies in which investigations were carried out on human beings or animals, as well as in clinical studies, approval of the Bioethics Committee must be obtained and relevant information be included in the manuscript.
6. An abstract of 200-250 words should precede the main article. Abstracts in English and in Polish should contain the following structure: aim, material and methods, results, conclusions. Abbreviations should not be used in the title or the abstract.
7. Key words (3-6) will be printed both in English and in Polish, according to the Medical Subject Heading Index Medicus Catalogue (MeSH) website http://www.nim.nih.gov.mesh/MBrower.html) Key words should not be a repetition of the title.
8. Illustrative material may include clear black and white or colour photographs on glossy paper, or clearly contrasting, carefully executed technical drawings on white paper. Photographs must be scanned on CD-ROM. On the back of each figure, please mark the top, and write the name of the author, title of the article and number of the figure. Captions for the figures should also be provided on a separate page in Polish and English. The text in the figures should also be both in Polish and in English.
9. Tables, their titles and content should be written both in English and Polish in Word for Windows in A4 format, with double spacing. All the footnotes referring to each table should be placed underneath the text of the table. Please mark on the margin of the text where the table or figure should be inserted.
10. References should be given in the order of citation and should be limited to those quoted in the text. In original articles, the number of references should not exceed 30, while in review papers 40. Each reference should include the surname(s) of the author(s) and the first letter of their first name(s), the title of the article, the abbreviated title of the journal (according to Index Medicus), the year, and number of the first and last page. When quoting book references, give the surname of the author(s), the first letter of the first name, the title of the chapter, the title of the book, the publisher, the place and year of publication. It is allowed to cite Internet references with the URL, with the date when the article was used and, if possible, the names of the authors.
11. After the references, the surname and first name, correspondence address, telephone number and e-mail address of the first author should be given.
12. The submitted manuscript must include a statement signed by all the authors describing the impact of each individual in preparing the article (concept and plan of the work, data collection and analysis, responsibility for statistical analysis, writing the article, critical review, etc.) including a declaration they take full responsibility for its contents. The statement must also include the assurance that the contents have not been published or sent for publication in other journals.
13. The authors also state that there is no conflict of interests at the time of submission, such as:
  – financial (employment, counseling, paid reviews, stock ownership, remuneration, etc.)
  – personal links
  – academic competition or other competition that can have an influence on the content of the paper
  – sponsoring of research or other studies involved in the submitted paper at the stage of designing research, collecting and interpreting data, or writing a report.
    A conflict of interests takes place when one or more authors have links of financial dependence with industry (through investment, employment, owning stocks or remuneration), directly or through the immediate family.
    If the work concerns products sponsored by a firm, the authors must include this information.
14. Submitted papers are peer-reviewed by two independent reviewers and are then qualified for publication by the Editor in Chief. Reviews are anonymous. The authors receive each reviewer’s comments concerning corrections or disqualification. The review procedure follows the recommendations of the Ministry of Science and Higher Education published as “Good Practice in Review Procedures in Science” (Warsaw 2012) and was described in detail on the www.medwiekurozwoj.pl website.
15. The editing board reserves the right to make relevant editing corrections.
16. Accepting the article for publication means that the copyright of the papers belongs to the editors of *Developmental Period Medicine*.
17. The authors receive one copy of the journal free of charge. On request, access to the PDF file containing their article is available.
18. Manuscripts not concordant with the above instructions will be returned to be corrected.
19. The editors do not return papers which have not been commissioned.
20. The editors take no responsibility for the contents of the advertisements.
